# Patient profiled data for treatment decision-making: valuable as an add-on to hepatitis C clinical guidelines?

**DOI:** 10.1186/s12911-024-02608-x

**Published:** 2024-08-13

**Authors:** Sylvia M. Brakenhoff, Thymen Theijse, Peter van Wijngaarden, Christian Trautwein, Jonathan F. Brozat, Frank Tacke, Pieter Honkoop, Thomas Vanwolleghem, Dirk Posthouwer, Stefan Zeuzem, Ulrike Mihm, Heiner Wedemeyer, Thomas Berg, Solko W. Schalm, Robert J. de Knegt

**Affiliations:** 1 Department of Gastroenterology and Hepatology, Erasmus MC, University Medical Center, Rotterdam, The Netherlands; 2 Department of Internal Medicine, Amphia Hospital, Breda, the Netherlands; 3 Leibnitz Institut Fuer Arbeitsforschung, Formerly Department of Internal Medicine III, University Hospital RWTH Aachen, Aachen, Germany; 4 Department of Hepatology & Gastroenterology, Charité University Medical Center, Berlin, Germany, formerly Department of Internal Medicine III, University Hospital RWTH Aachen, Aachen, Germany; 5 Department of Hepatology and Gastroenterology, Charité - Universitätsmedizin Berlin, Campus Virchow-Klinikum (CVK) and Campus Charité Mitte (CCM), Berlin, Germany; 6 Department of Gastroenterology and Hepatology, Albert Schweitzer Hospital, Dordrecht, the Netherlands; 7 Department of Gastroenterology and Hepatology, Antwerp University Hospital, 2650 Antwerp, Belgium; 8 Viral Hepatitis Research Group, Laboratory of Experimental Medicine and Pediatrics, University of Antwerp, 2650 Antwerp, Belgium; 9 Department of Internal Medicine, Division of Infectious Diseases, Maastricht University Medical Centre, Maastricht, the Netherlands; 10 Department of Medical Microbiology, Division of Infectious Diseases, Maastricht University Medical Centre, Maastricht, the Netherlands; 11 Department of Internal Medicine 1, Goethe University Hospital, Frankfurt Am Main, Germany; 12 Department of Gastroenterology, Hepatology and Endocrinology, Hannover Medical School, Hannover, Germany; 13 Division of Hepatology, Department of Medicine II, Leipzig University Medical Center, Leipzig, Germany; 14 TherapySelector, Rotterdam, the Netherlands

**Keywords:** Personalized medicine, Patient profiles, Guidelines, Hepatitis C, Data-driven medicine

## Abstract

**Background and Aims:**

Systematic reviews and medical guidelines are widely used in clinical practice. However, these are often not up-to-date and focussed on the average patient. We therefore aimed to evaluate a guideline add-on, TherapySelector (TS), which is based on monthly updated data of all available high-quality studies, classified in specific patient profiles.

**Methods:**

We evaluated the TS for the treatment of hepatitis C (HCV) in an international cohort of patients treated with direct-acting antivirals between 2015 and 2020. The primary outcome was the number of patients receiving one of the two preferred treatment options of the HCV TS, based on the highest level of evidence, cure rate, absence of ribavirin-associated adverse effects, and treatment duration.

**Results:**

We enrolled 567 patients. The number of patients treated with one of the two preferred treatment options according to the HCV TS ranged between 27% (2015) and 60% (2020; *p* < 0.001). Most of the patients received a regimen with a longer treatment-duration (up to 34%) and/or addition of ribavirin (up to 14%). The effect on the expected cure-rate was minimal (1–6% higher) when the first preferred TherapySelector option was given compared to the actual treatment.

**Conclusions:**

Medical decision-making can be optimised by a guideline add-on; in HCV its use appears to minimise adverse effects and cost. The use of such an add-on might have a greater impact in diseases with suboptimal cure-rates, high costs or adverse effects, for which treatment options rely on specific patient characteristics.

**Supplementary Information:**

The online version contains supplementary material available at 10.1186/s12911-024-02608-x.

## Introduction

Due to the rapid increase in publications that comprise factual medical knowledge, only a limited number of physicians can be completely up-to-date, including opinion leaders and those who perform systematic reviews or generate medical guidelines.

Systematic reviews include relevant literature on a specific topic that has been published until that moment, using highly sensitive search engines of (bio)medical literature databases. Such a strategy identifies hundreds to thousands of publications, of which approximately 1% will be included in the final systematic review [1, 2]. This method is time-consuming and results in a review that can be outdated at the time of publication [3]. The problem of keeping up to date is also observed in medical guidelines. In addition, medical guidelines comprise a substantial number of pages, making them time-consuming to access. Finally, guidelines are generated by a small expert panel and can be considered to some extent subjective [4]. Netherlands Organisation for Health Research and Development has called for innovative guideline add-ons that mitigate the problems of guidelines [5].

Such an add-on guideline should provide reliable and up-to-date information regarding treatment outcomes of a disease of interest, accessible for every physician and patient. This calls for a large dataset obtained from high-quality studies, including the treatment response rate of all available medicines approved by the Food and Drug Administration (FDA) and/or European Medicine Agency (EMA) [6, 7]. Such a database has been developed and tested to treat viral hepatitis C infection: HCV TherapySelector (HCV TS).

The HCV TS has been introduced in clinical practice in the Netherlands. In this study, we aimed to assess the added value of the TS in real practice.

## Methods

### The TherapySelector

The TherapySelector (TS) displays patient profiled data from high-quality publications on pharmacotherapy of a specific disease via a mobile or web-based application [8]. The application provides information about the efficacy, adverse effects and costs of published treatment regimens of licensed drugs for an individual patient based on his/her specific patient profile. With a monthly update, this application can offer more up-to-date information than guidelines.

For the Hepatitis C module of TS, patient profiles include one patient characteristic of each of four categories: HCV genotype (1–6), disease stage (acute hepatitis, chronic hepatitis without cirrhosis, chronic hepatitis with compensated cirrhosis, and chronic hepatitis with decompensated cirrhosis), treatment status (treatment naïve, pegylated interferon (PEG-IFN) experienced, sofosbuvir experienced, or NS5A + NS3 experienced) and comorbidities (HCV-HBV co-infection, HCV-HIV co-infection, liver transplantation, renal failure [eGFR < 30], none of above). Per patient profile, the TS displays the different treatment options, defined by therapy names, daily dose, and duration (therapy regimen). Per patient profile-therapy regimen, it shows the expected cure rate, level of evidence (high – medium – low evidence), price of treatment and reimbursement, side effects, and possible drug-drug interactions. The level of evidence was based on the number of studies and number of participants per profile.

With these definitions a search string has been developed to identify relevant medical literature in the MEDLINE database PubMed. As the search is repeated every month, much effort was devoted to creating a search string with focus on high specificity and acceptable sensitivity. In Supplementary File 1, the components and development of this search string are described in detail. The data of these studies is obtained directly from the published article or requested from the corresponding author. The data collected is stratified based on specific patient profiles (Supplementary File 2) and as such used as input for the TS application (Supplementary File 3). Herewith, a physician can select an evidence-based treatment option that is personalized for a specific patient.

### Study design, population, and data acquisition

We conducted an international, multicentre observational cohort study, including 16 sites from the Netherlands, Belgium, and Germany. Data was obtained of consecutive patients aged > 18 years, with a chronic HCV mono-infection, who received direct-acting antiviral (DAA) treatment between 2015 and 2020. Data was collected, including patient-, virus-, liver disease stage -, and therapy- related characteristics as well as the outcome of HCV infection.

Patient factors included age, sex, and ethnicity. Viral factors included genotype and HCV RNA viral load; disease stage were liver fibrosis stage and Child Pugh score in case of liver cirrhosis. Therapy-related factors related to previous therapy and DAA regimen defined by drug names, addition of ribavirin and duration. Comorbidities included dialysis, liver transplantation, or hepatocellular carcinoma (HCC). Curation was defined as achievement of undetectable HCV RNA 12–24 weeks after end of DAA therapy (i.e. sustained virological response; SVR). Diagnosis of liver cirrhosis was based on ultrasound findings compatible with cirrhosis, histology, or a liver stiffness measurement of ≥ 12.5 kPa (using transient elastography).

### Endpoints

The primary endpoint was the number of patients that received one of the two preferred treatment options of the HCV TS. Secondary outcomes included the number of patients that received a treatment regimen with a lower level of evidence than recommended by the HCV TS. In addition, we assessed the number of patients that received one of the two preferred treatment options of the EASL guideline. Finally, we compared the preferred treatment options of the HCV TS with those of the EASL guideline with regard to efficacy, ribavirin addition and costs.

### Determination of preferred treatments

**Fig. 1:**
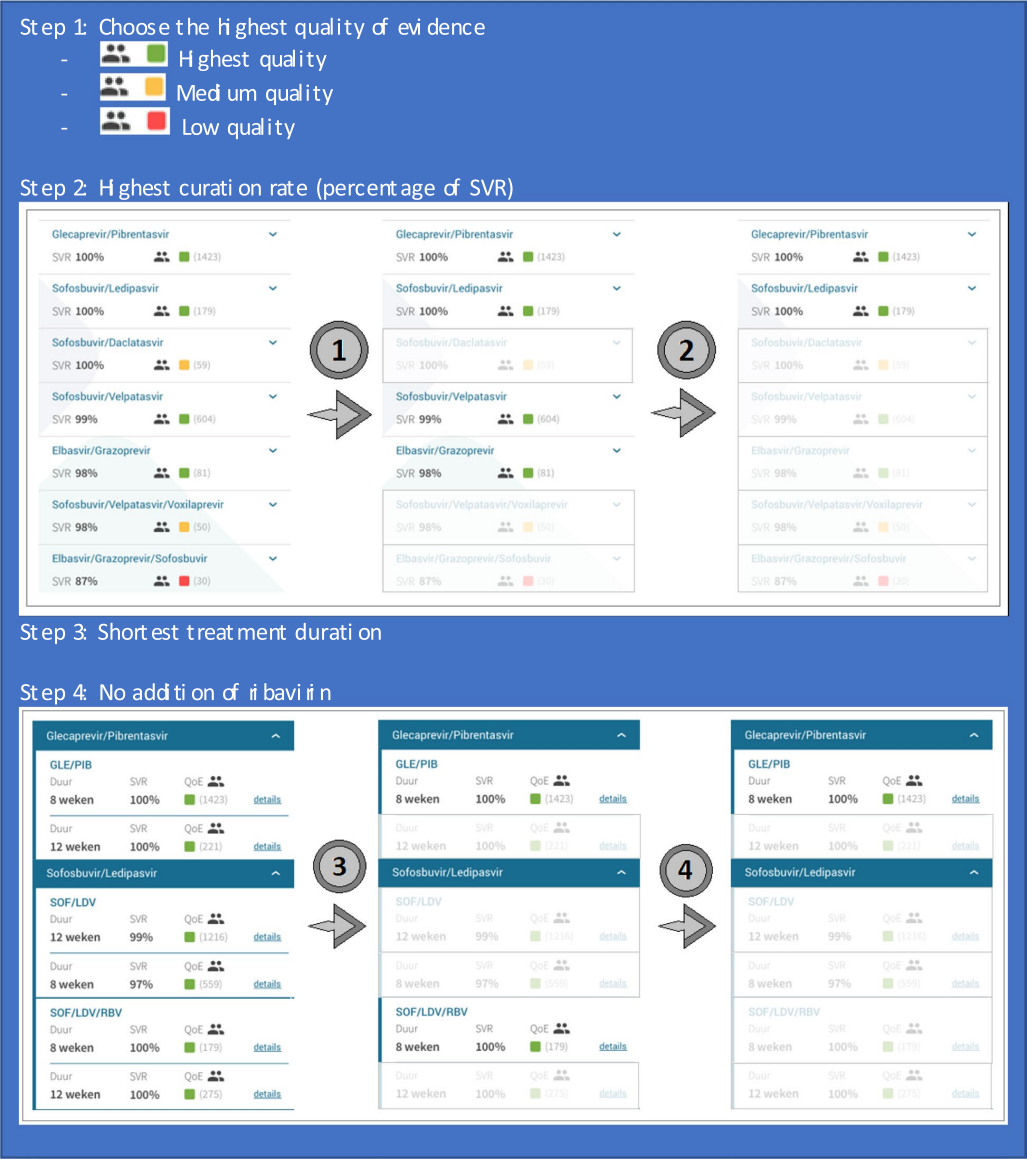
Process of determination of the preferred treatment option using the HCV TS. Abbreviations: *HCV* Hepatitis C virus, *SVR* Sustained virological response, *TS* TherapySelector

For each patient profile, the preferred treatment options were established by two independent researchers (SB, TT). In case of incoherence, a senior researcher and viral hepatitis expert was consulted (RdK). The preferred treatment options we established for various patient profiles were based on consecutively the highest level of evidence (based on the number of patients of a specific patient profile included in the TS), highest cure rate (percentage of SVR), shortest treatment duration, and no addition of ribavirin. Per patient profile, we assessed both the HCV TS and EASL guideline to select the two preferred treatment options, stratified on year of treatment. Figure 1 displays the process of determination of the preferred treatment option. For the HCV TS, we used the first application version that was available in each year. For the EASL guideline, we assessed the version available in the year of DAA treatment (EASL 2015 [9] for the year 2015, EASL 2016 [10] for the years 2016–2017, EASL 2018 [11] for the years 2018–2020).

### Statistical analysis

For each patient included in this study, a treatment regimen as defined by the TS has a name, dosage, and duration. We assessed whether the treatment prescribed was in line with the first or second treatment option of the HCV TS and EASL guideline respectively.

Next, using the HCV TS, we calculated the level of evidence and expected cure rate of the prescribed DAA regimen in real practice. Then we compared the predicted level of evidence and cure rate of the first preferred treatment option according to the HCV TS and the prescribed DAA regimen. Data were stratified by year of DAA treatment. Descriptive data was reported using percentages, means (± SD) or medians (IQR) as appropriate. Data was tested for significance using chi-squared test. Statistical analysis was performed using IBM SPSS for Windows version 28.0 (SPSS Inc., Chicago, Illinois, USA). Graph Pad Prism version 8 for Windows (GraphPad Software, San Diego, California, USA) was used for graphical representation of the results.

## Results

### Study population

In total, we enrolled 567 patients from 16 different sites in the Netherlands, Belgium, and Germany. Patient characteristics are displayed in Table 1. The majority of patients had HCV genotype 1 (53%) or genotype 3 (26%), and were not previously treated with other antiviral agents (i.e. treatment naïve; 76%). Liver cirrhosis was present in 118 patients (21%), of whom 15 patients (13%) had Child Pugh B/C liver cirrhosis. Sustained virological response (SVR) was achieved in 542 patients (96%).

**Table 1:** Patient characteristics

N,%	**2015** **N = 22**	**2016** **N = 67**	**2017** **N = 81**	**2018** **N = 116**	**2019** **N = 181**	**2020** **N = 100**
**HCV genotype**						
Genotype 1a/b/other	2/11/0 (9.1/50.0/0)	24/18/1 (35.8/26.9/1.5)	23/16/3 (28.4/19.8/3.7)	28/29/3 (24.1/25.0/2.6)	36/52/4 (19.9/28.7/2.2)	31/19/0 (31.0/19.0/0.0)
Genotype 2	2 (9.1)	9 (13.4)	6 (7.4)	5 (4.3)	10 (5.5)	2 (2.0)
Genotype 3	3 (13.6)	11 (16.4)	20 (24.7)	31 (26.7)	56 (28.7)	25 (25.0)
Genotype 4	4 (18.2)	3 (4.5)	7 (8.6)	14 (12.1)	10 (5.5)	7 (7.0)
Genotype 5/6	0 (0)	0 (0)	1 (1.2)	0 (0)	1 (0.6)	0 (0)
Unkown	0 (0)	1 (1.5)	5 (6.2)	6 (5.2)	12 (6.6)	16 (7.0)
**Liver cirrhosis**	11 (50.0)	12 (17.9)	11 (13.6)	23 (19.8)	37 (20.4)	24 (24.0)
Child Pugh A	9 (81.8)	10 (83.3)	8 (72.7)	19 (82.6)	29 (78.4)	19 (79.2)
Child Pugh B/C	0 (0)	1 (8.3)	2 (18.2)	3 (13.0)	5 (13.5)	4 (16.7)
Unknown	2 (18.2)	1 (8.3)	1 (9.1)	1 (4.3)	3 (8.1)	1 (4.2)
**Comorbidities**						
Chronic kidney disease	1 (4.5)	2 (1.5)	0 (0)	3 (2.6)	1 (0.6)	0 (0)
**Treatment status**						
Treatment naïve	8 (36.4)	45 (67.2)	46 (63.0)	92 (79.3)	150 (82.9)	83 (83.0)
PEG-IFN-experienced	13 (59.1)	18 (26.9)	23 (31.5)	15 (12.9)	20 (11.0)	12 (12.0)
DAA-experienced	1 (4.5)	4 (6.0)	4 (5.5)	9 (7.8)	11 (6.1)	5 (5.0)
**DAA regimen**						
ELB/GRZ	-	-	5 (6.2)	11 (9.5)	29 (16.0)	8 (8.0)
GLE/PIB	-	-	-	46 (39.7)	76 (42.0)	69 (69.0)
OMV/PTV/RTV + DSV	5 (22.7)	7 (10.4)	3 (3.7)	-	-	-
SOF	4 (18.2)	2 (3.0)	-	1 (0.9)	-	-
SOF/DCV	7 (31.8)	27 (40.3)	25 (30.9)	6 (5.2)	-	-
SOF/LDV	4 (18.2)	30 (44.8)	32 (39.5)	15 (12.9)	2 (1.1)	-
SOF/SIM	2 (9.1)	-	1 (1.2)	-	-	-
SOF/VEL	-	-	14 (17.3)	39 (29.3)	61 (33.7)	19 (19.0)
SOF/VEL/VOX	-	-	1 (1.2)	1 (0.9)	12 (6.6)	3 (3.0)
SOF + GLE/PIB	-	-	-	2 (1.7)	-	1 (1.0)
Other	-	1 (1.5)	-	-	1 (0.6)	-
**Addition ribavirin**	14 (63.6)	10 (14.9)	8 (9.9)	2 (1.7)	4 (2.2)	3 (3.0)
**SVR**	21 (95.5)	64 (95.5)	78 (96.3)	113 (97.4)	173 (96.6)	93 (93.0)

*HCV* Hepatitis C virus*, PEG-IFN* Pegylated interferon*, DAA* Direct-acting antivirals, *ELB* Elbasvir, *GRZ* Grazoprevir, *GLE* Glecaprevir*, PIB* Pibrentasvir, *OMV* Ombitasvir, *PTV* Paritaprevir*, RTV* Ritonavir, *DSV* Dasabuvir, *SOF Sofosbuvir, DCV* Daclatasvir, *LDV* Ledipasvir, *SIM* Simeprevir*, VEL* Velpatasvir, *VOX* Voxilaprevir*, SVR* Sustained virological response

### Comparison of the actual treatment options and the preferred treatment options according to the Therapy Selector

**Fig. 2:**
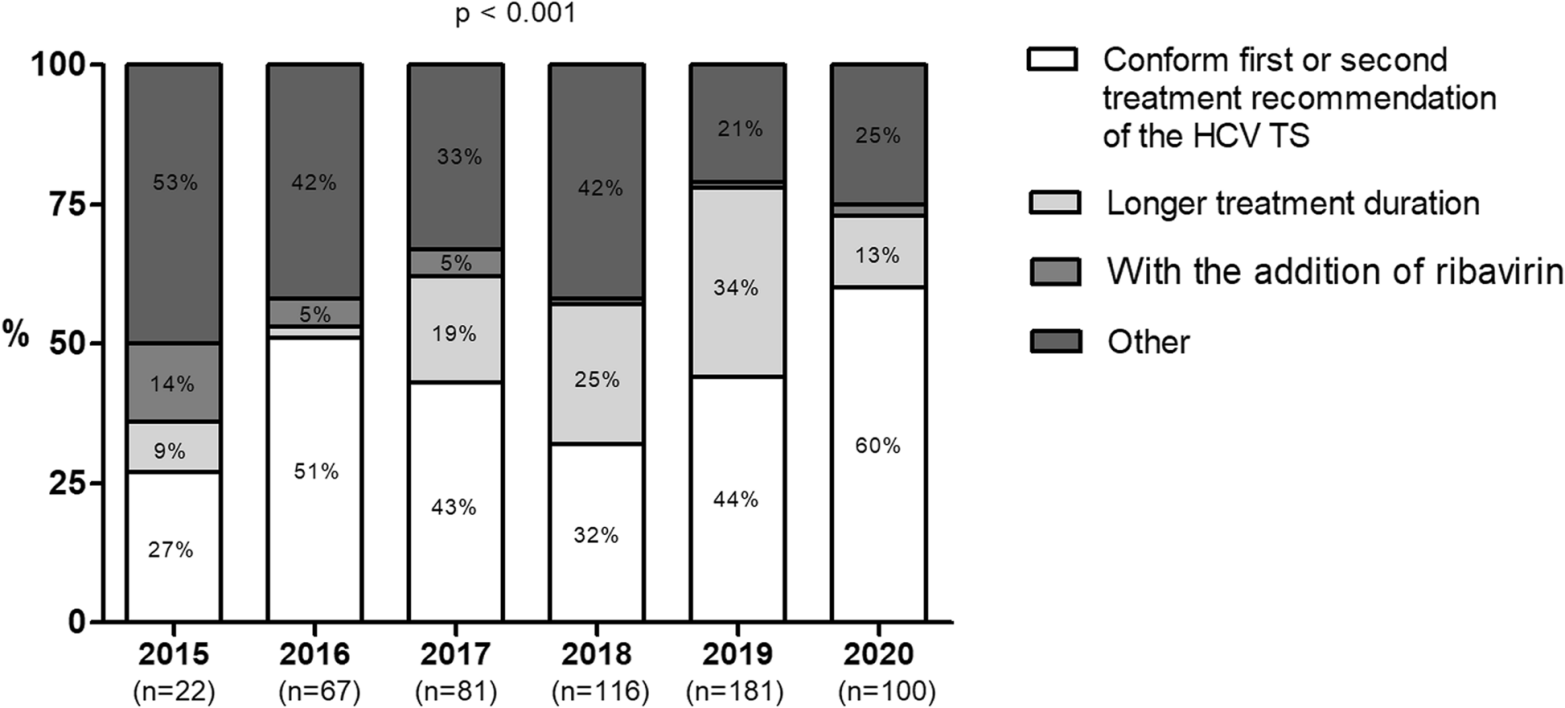
Rates of patients that received the same or different treatment regimen according to the preferred treatment options of the HCV TS, stratified by year of DAA treatment. Abbreviations: *HCV TS* Hepatitis C TherapySelector

The number of patients that have been treated with one of the two preferred treatment options according to the HCV TS ranged between 27% (2015) and 60% (2020; *p* < 0.001, Fig. 2). Among the patients that received a different treatment regimen compared to the preferred DAA agents in the HCV TS, most patients received a regimen with a longer treatment duration, or a different DAA agent but with the similar treatment duration and ribavirin regimen (Fig. 2).

Among the patients that received different treatment regimes, the actual treatment option was of a lower level of evidence compared to the DAA options preferred by the HCV TS in 8% (2020) to 27% (2015) of the patients (Table 2). Among patients treated with one of the two preferred treatment options according to the HCV TS, we did not observe a higher number of successfully treated patients (i.e. patients that achieved SVR; *p* > 0.050) compared to patients that did not receive one of the preferred treatment options.

**Table 2:** Comparison of the level of evidence

	**2015** **N = 15**	**2016** **N = 32**	**2017** **N = 46**	**2018** **N = 76**	**2019** **N = 102**	**2020** **N = 40**
**Lower level of evidence of real treatment option** (n, %)	4 (26.7)	8 (25.0)	6 (13.0)	19 (25.0)	11 (10.8)	3 (7.5)
**Difference in SVR% (among patients with same level of evidence)** (median, range)	3 (3–6)	2 (1–6)	3 (1–4)	1 (1–3)	1 (1–3)	1 (1–2.5)

Comparison of the level of evidence between real treatment regimen and preferred treatment option according to the HCV TS, among patients that received another treatment regimen than the preferred treatment options according to the HCV TS

*SVR* Sustained virological response, *HCV TS* Hepatitis C Therapy Selector

Among the patients treated with a level of evidence equal to the option preferred by the HCV TS, the expected SVR probability was 1–6% higher when the first preferred treatment option in the HCV TS was given compared to the actual treatment (Table 2).

### Comparison real treatment versus EASL guideline

**Fig. 3:**
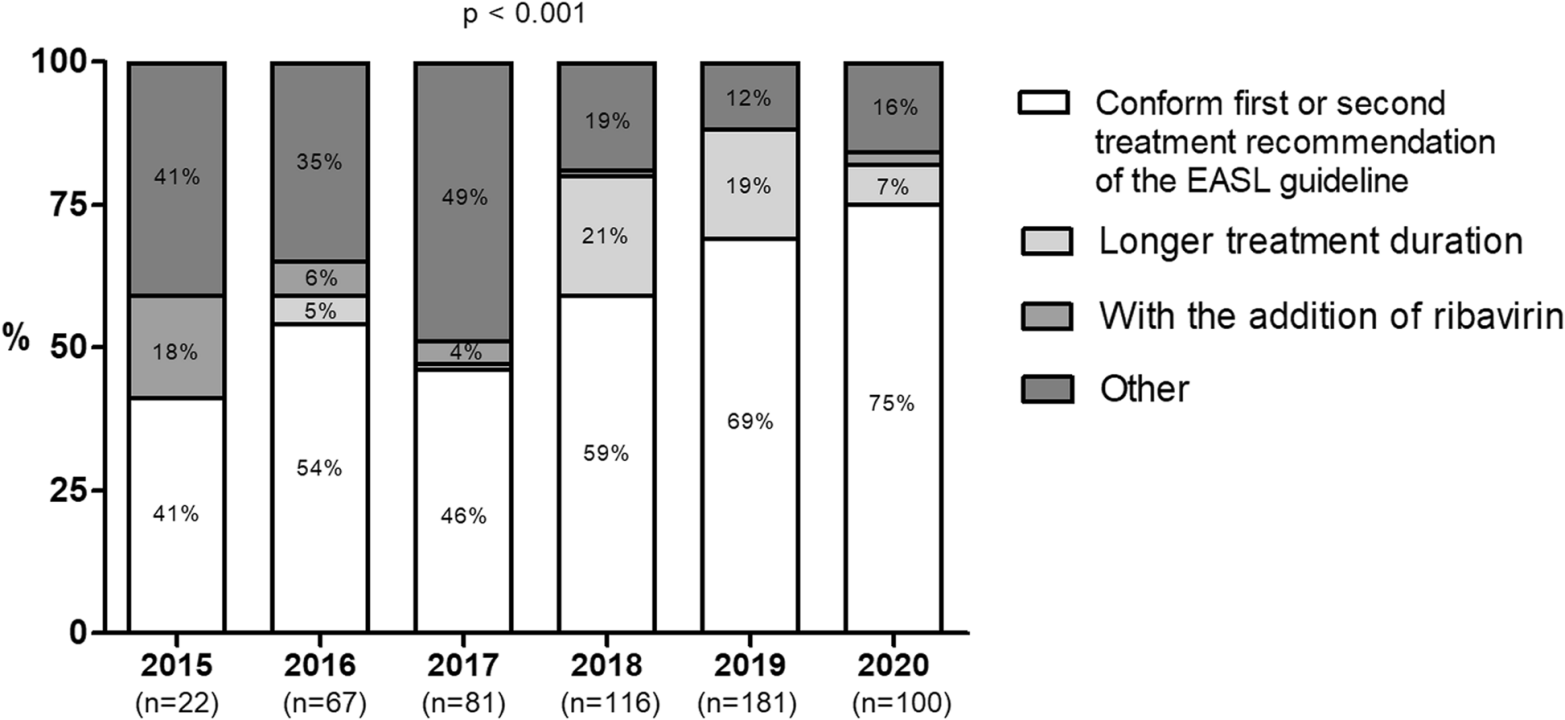
Rates of patients that received the same or different treatment regimen according to the preferred treatment options of the EASL, stratified by year of DAA treatment. Abbreviations: *EASL* European Association for the Study of the Liver

The number of patients that were treated with one of the two preferred treatment options according to the EASL guideline ranged between 41% (2015) and 75% (2020; *p* < 0.001, Fig. 3).

In addition, the preferred treatment options according to the HCV TS matched in 66% to 91% of the patients with at least one of the preferred treatment options according to the EASL guidelines (Table 3).

**Table 3:** Similarities in preferred treatment options between EASL and HCV TS

	**2015** **N = 21**	**2016** **N = 67**	**2017** **N = 80**	**2018** **N = 114**	**2019** **N = 181**	**2020** **N = 100**
**No match** (n, %)	5 (23.8)	13 (19.7)	27 (33.8)	14 (12.3)	17 (9.4)	16 (16.0)
**1 match** (n, %)	14 (66.7)	48 (72.7)	39 (48.8)	95 (83.3)	158 (87.3)	80 (80)
**2 matches** (n, %)	2 (9.5)	5 (7.6)	14 (17.5)	5 (4.4)	6 (3.3)	4 (4.0)

*EASL* European Association for the Study of the Liver, *HCV TS* Hepatitis C Therapy Selector

## Discussion

In this study, we evaluated the use of an add-on guideline: the HCV Therapy Selector (TS). Using a large international cohort, we demonstrate that a significant part of the DAA-treated patients with a chronic hepatitis C infection did not receive a treatment regimen based on the preferred treatment options of the HCV TS. The use of the HCV TS could theoretically have resulted in treatment options with a higher cure rate, a lower adverse effect rate and/or less cost. Our results point to the main effect of using HCV TS as a reduction of up to 34% in treatment duration with associated lower costs and a somewhat smaller reduction in the use of ribavirin with its associated adverse effects.

Are the results likely to be true or influenced by the selection of patients in the study, the availability of drugs at the time of prescription or restrictions for payment. Modern hepatitis C is so effective that it will be difficult to document an increase in efficacy in a usual hepatitis C population. If the study had been restricted to patient with cirrhosis and patients who had previously failed DAA therapy, a clinically relevant effect on efficacy would still be possible. An effect of limited availability of new drugs or restrictions for payment on our results is unlikely as availability and reimbursement of DAA in the Netherlands has lagged those in Germany and Belgium.Current medical practice is based on evidence-based decision-making. However, an individual physician cannot be entirely up to date, considering the large number of studies that are published daily. Therefore, a small selection of experts reviews the available literature often already including systematic reviews and meta-analyses and summarises it into medical guidelines. However, those reports are increasingly criticized because they are often outdated and time-consuming to access [4, 5, 12]. Therefore, a more up-to-date, more accessible and more personalised alternative or add-on is needed.

In contrast to guidelines, the TS is based on the original data from the studies included in the meta-analyses and systematic reviews of guidelines; it also includes original data from high-quality prospective cohort studies with real-world data to fill the gap created by the selection criteria of many randomised controlled trials. As soon as a new study is published (usually epub) TherapySelector tries to include the new data in the database, thereby minimising the time lag of clinical guidelines. By using patient profiles, formed by four patient characteristics, accessibility to the combined original data is strongly enhanced. Percentage disease remission/cure, percentage adverse effects and cost are presented in one overview. The physician sees the results of all published treatment regimens in terms of efficacy, adverse effects and cost and can select the evidence-based option that fits his patient best. This approach appears particular useful for patients at risk of considerable morbidity of adverse effects or when reimbursement or availability of guideline recommended medication is a problem.

Using the HCV TS, we show in this study that the preferred treatment options given by the HCV TS corresponded in the majority of patient profiles with the EASL guideline. However, our results suggest that the curation rate could be 1–6% higher than the treatment options given in real life. In addition, we observed that many patients were treated with a longer treatment duration and/or with the addition of ribavirin, which is associated with higher drug costs and/or side effects.

Since 2014, treatment results for viral hepatitis C have improved impressively. Whereas the treatment options were considered complex in 2015, nowadays treatment options include a few highly effective pan-genotypic DAA agents, making the treatment for chronic hepatitis C simpler and less affected by specific patient characteristics. This could have caused the progressive reduction in the differences between the DAA therapy given and preferred DAA treatment options according to the HCV TS. Still, even in 2020, 40% of therapies given were not among the two treatment options preferred by TS. Therefore, the TS might be an important and valuable device for diseases with multiple treatment options of variable curation rates, which are affected by different patient characteristics. Some examples might include hypertension, HIV, and hepatitis B.

What is more, the TS can be a valuable tool to facilitate access to antiviral therapy and increase the numbers of patients who receive treatment for hepatitis C at all. After being diagnosed with chronic hepatitis C, a relevant number of patients gets lost before receiving treatment from a specialist doctor. A tool like the TS could provide general doctors or other non-specialist health care providers, for instance in addiction centers or prisons, with the help they need to treat patients themselves, directly after the detection of a chronic hepatitis C infection, thus increasing the rates of actually treated patients.

Although our findings are based on a large international cohort, several limitations should be acknowledged. First, our cohort includes predominantly non-cirrhotic (79%), treatment naïve patients with HCV genotype 1 (53%). Those patients can be considered “easy to cure”. Possibly, HCV TS might have more value among more challenging patients to treat, such as patients with liver cirrhosis. Also, the preferred treatment options were selected by two independent researchers, thus a certain degree of subjectivity cannot be ruled out. Next, HCV treatment includes agents with a very high curation rate (> 93% in our cohort), making it difficult to document an added value of the use of TS. Therefore, our findings warrant confirmation in diseases with lower controllable rates, such as hypertension and heart failure. In this study, it was not possible to make a comparison with the EASL HCV advisor application, as the EASL is no longer distributing, updating or supporting this application anymore.

## Conclusion

In conclusion, the Therapy Selector might serve as an instrument to guide medical decision making for individual patients. The clinical implications of such an add-on guideline might be increased efficacy and diminished adverse events or costs, all valuable in diseases for which the treatment options are affected by specific patient characteristics. The results of this study should be confirmed for other diseases.

### Supplementary Information

Supplementary Material 1.

## Data Availability

All data generated or analysed during this study are included in this published article.

## Abbreviations

FDA: Food and Drug Administration
EMA: European Medicine Agency
HCV: Hepatitis C virus
EASL: European Association for the Study of the Liver
TS: Therapy Selector
PEG-IFN: Pegylated interferon
SVR: Sustained virological response
DAA: Direct-acting antiviral
