# Supplementary material for: Patient profiled data for treatment decision-making: valuable as an add-on to hepatitis C clinical guidelines?
Source: BMC Med Inform Decis Mak. 2024 Aug 13;24:227. doi: 10.1186/s12911-024-02608-x (PMC11321176; doi:10.1186/s12911-024-02608-x)
Supplement: Supplementary file 1 — Supplementary Material 1. [file 12911_2024_2608_MOESM1_ESM.docx]

**Supplementary file 1**

PubMed search string

The PubMed search string comprises multiple key words, stratified into four components. These components include (1) disease domain, (2) high-quality studies, (3) FDA/EMA approved medication, and (4) outcome measure. First, disease domain includes a specific disease/illness or therapeutic area of interest. The second component, high-quality studies, includes phase 2 or phase 3 (randomised controlled) clinical trials or prospective phase 4 studies (so called “real-life” studies). The third component includes FDA (Food & Drug Administration^1^), EMA (European Medicines Agency^2^) or MEB (Medicine Evaluation Board^3^) approved agents for the disease. Finally, the fourth component, outcome measure, includes cure or disease remission, whatever is applicable as relevant short term treatment outcome for the disease of interest.

For each component, specific key words were selected and tagged with search term qualifiers [title] for Title or [tiab] for Title and Abstract. Several search terms were combined using the linking word “OR” to increase sensitivity. To limit non-relevant articles as much as possible, additional search terms were added using the linking word “NOT”. Finally, the cluster of search terms per component were joined using the linking word “AND”.

In general, each component started with a sensitive search term (preferably a MeSH term^4^). Since MeSH terms are not assigned immediately by PubMed after publication of an article, more specific search terms were added in combination with [title] or [tiab]. The asterisk (*) was used on terms that could contain multiple sub-terms, for instance articles using the terms ‘randomised’ and ‘randomized’ could both be included using the term ‘randomi*’.

To check the sensitivity, the search string was tested in an alternative biomedical literature database Embase. In addition, references of international guidelines for the management for HCV were reviewed and compared with the studies obtained by the search string. Relevant studies that were missed by the PubMed search string were included in the TS database and also used to optimise the PubMed search string.

The effect on the number of identified publications was studied after each adjustment of the search. The aim was to develop a search string that identified a large number of relevant publications with as little as possible non-relevant articles. Search terms in order to exclude non-relevant articles were combined with the linking word “NOT” and [title] to prevent excluding relevant articles.

Although the search string was initially developed for hepatitis C, and comprises HCV-specific key words, the search string is in design generally applicable/adaptable for other diseases.

Assessment of the specificity and sensitivity.

The search string for HCV identified 88 studies in the period between 01-01-2019 and 31-12-2019. After screening on title and abstract, 68 articles (77%) were considered as relevant high-quality studies.

For the sensitivity assessment, the references of international guideline were studied.^5^ This guideline included 93 high-quality publications assessing the efficacy of DAAs. The search string was tested in the DAA period (2014-2019), and identified 76/93 articles (82%). Among the 17 references that were not identified by the search string, seven references included abstracts submitted to international conferences who were never published.

In addition, we assessed a recently published systematic review. *^6^* Of the 28 articles that were included in the systematic review, 14 articles (50%) were also identified by the search string. The other 14 articles (50%) included studies using DAA agents that were not (yet) approved by the FDA or EMA.

**Supplementary Table 1. Search string hepatitis C, April 2020**

| **Components search string** | **Key words** |
| --- | --- |
| Disease domain | (Hepatitis C[MeSH] OR hepatitis C[tiab] OR HCV[tiab]) |
| High-quality studies | AND ((Randomized Controlled Trial [Publication Type] OR randomi*[tiab] OR phase 2*[tiab] OR phase 3*[tiab] OR phase II*[tiab] OR phase III*[tiab]) OR ((real-world[tiab] OR real-life[tiab] OR observational cohort[tiab] OR phase 4*[tiab] OR phase IV*[tiab] OR open label[tiab] OR "Clinical trial number"[tiab]) AND (prospectiv*[tiab] OR consecutiv*[tiab] OR registry[tiab] OR intent-to-treat[tiab] OR "Clinical trial number"[tiab]))) NOT (Review[Publication type] OR Review*[tiab] OR Case report*[tiab] OR retrospectiv*[tiab] OR integrated analy*[tiab] OR pooled data[tiab] OR study protocol[title] OR design[title] OR analysis[title] OR case*[title] OR reply[title]) |
| FDA-EMA approved agents | AND ((paritaprevir[tiab] OR ABT-450/r[tiab]) OR grazoprevir[tiab] OR (glecaprevir[tiab] OR ABT-493[tiab]) OR simeprevir[tiab] OR (voxilaprevir[tiab] OR GS-9857[tiab]) OR ombitasvir[tiab] OR elbasvir[tiab] OR daclatasvir[tiab] OR ledipasvir[tiab] OR velpatasvir[tiab] OR (pibrentasvir[tiab] OR ABT-530[tiab]) OR (sofosbuvir[tiab] OR GS-7977[tiab]) OR Dasabuvir[tiab] OR DAA*[title] OR direct acting antiviral*[title] OR Interferon-free [title]) NOT (ruzasvir[title] OR uprifosbuvir[title] OR odalasvir[title] OR asunaprevir[title] OR telaprevir[Title] OR boceprevir[Title] OR danoprevir[Title] OR vaniprevir[Title] OR alisporivir[Title] OR beclabuvir[Title] OR fluvastatin[Title] OR faldaprevir[Title] OR mericitabine[Title] OR balapiravir[Title] OR nitazoxanide[Title]) |
| Clinical outcome | AND (remission induction[mesh] OR cure*[tiab] OR sustained virologic* [tiab] OR SVR*[tiab]) NOT (diagnostic accuracy[tiab] OR patient-reported outcomes[tiab] OR PROM[tiab] OR cost-effectiveness[tiab] OR immune response*[title] OR quality of life[title] OR reinfection*[title] OR kinetic*[title] OR pharmacokinetic*[title] OR risk factor*[title] OR predict*[title]) |

**Supplementary file 2**

Patient profiles

How can a guideline ‘add-on’ provide more personalized or person-oriented information than a guideline? When the term personalized medicine was introduced, the concept was based on a dominant role of an individual's genetic profile. Nowadays it appears that the predictive value of simple clinical information is at least as relevant.^7,8^ It therefore seems desirable to use not only genetic traits for person-oriented choice of therapy, but all proven prognostic and predictive factors.

The source of information for proven prognostic and predictive factors are publications of randomized controlled trials, clinical phase 2 and phase 3 studies and prospective real-life studies with IRB approval and < 10% non-outcome patients, with information on prognostic or predictive factors that affect the outcome of a therapy. To date, this information usually is based on subgroup analysis and identifies single variables.

However, when we think about how clinicians identify treatments suitable for a specific patient, they usually weigh more than one patient characteristic into that decision. They don’t ask “What treatment should I prescribe based on the patient’s sex?"; the correct question is "What treatment should I prescribe for this patient based on her age, sex, disease stage, previous therapies and comorbidities?". Kent and others have long been arguing for more nuanced subgroup analyses based on multiple variables rather than single-variable stratifications.^9^
Multiple-variable stratification using known prognostic and predictive factors that affect the outcome of a therapy yields so-called *patient profiles* that have a better fit for individual patients than the average.

If the guideline add-on’ is to be used in a busy physician's office, the number of patient characteristics that a physician must fill in must be limited. Prognostic and predictive factors are therefore modelled into 4 presumably causal categories: etiology-pathophysiology including genetic information, disease progression, previous therapy status and co-morbidity. There are multiple choices in each category.

Below, further details are given derived from the hepatitis C domain.

a. Etiology, pathophysiology including genetic information
Hepatitis C is caused by a virus. Outcome of therapy (SVR12=cure of infection) is affected by the virus genotype. There are 6 genotypes; each genotype has subtypes. Responses to early treatment combinations differed between genotype 1a and 1b. With therapies that were subsequently developed, differences in treatment outcome between subtypes disappeared but remained between genotypes. The most modern therapies show little differences in outcome between genotypes.
For hepatitis C the category Etiology initially had 6 choices: genotype 1-6. To be informative about the subtype effect of early therapies, results of those therapies were given according to subtype 1a and 1b.

b. Disease progression-activityIn general outcome of treatment is affected by the stage of the disease.
In hepatitis C, within the large group of chronic hepatitis C the presence of cirrhosis and within that group advanced or decompensated cirrhosis are factors associated with treatment failure. In acute hepatitis therapy response are according to current data not affected by patient baseline factors.

c. Previous therapy status
In many diseases outcome of treatment is affected by previous therapy status.
In hepatitis C interferon (IFN) or peginterferon (Peg-IFN) has been used from 2000 – 2014, with treatment failure rates over 50%. In 2014 sofosbuvir-based (SOF) combination therapy was introduced, and therapy failure dropped below 10%; shortly thereafter another class of direct-acting antivirals (DAA, NS5A-NS3 blockers) had similar therapy outcome.
Therapy outcomes are best in previously untreated patients, and -for most DAA- less in those with previous failed IFN therapy. Patients who previously failed NS5A-NS3 or SOF-based therapy will have poor outcomes with standard therapy regimens and require a different approach.

d. Co-morbidity
In real-life clinical practice many patients have co-morbidities. Not all co-morbidities affect the outcome of therapies, but some do.
Classifying a factor as co-morbidity or as etiology-pathophysiology is somewhat arbitrary; here we classify a factor as co-morbidity if the outcome assessment of the group defined by etiology/pathophysiology, disease stage and previous therapy is influenced by additional factors (for instance: renal failure: drug clearance plays a role; etcetera).

In hepatitis C co-infection with HIV was an important factor affecting outcome of interferon therapy; in the era of direct-acting antivirals this factor has become negligible. Renal failure is a separate group in view of variable antiviral drug metabolism and renal clearance; transplantation in view of potential drug interaction.

Person-oriented information in the TherapySelector is thus *patient profiled* information. From all the data about outcomes of therapy in a specific disease, only outcome data is presented from a specific patient profile that the user has selected. Each patient profile is defined by 4 prognostic and predictive factors, one from each category etiology-pathophysiology including genetic information, disease progression, previous therapy status and co-morbidity. For hepatitis C, 384 different patient profiles are possible.

**Supplementary Table 2. Patient profiles hepatitis C May 2020**

| **Patient profiles Hepatitis C** | **Number of choices** | **Calculation number of profiles** | **Total** |
| --- | --- | --- | --- |
| **Etiology-pathophysiology including genetics (virus genotype 1,2,3,4,5,6)** | 6 | 6x1 | = 6 |
| **Cirrhosis stage / disease activity (No, Child A, B/C; acute hepatitis)** | 4 | 4x6 | = 24 |
| **Previous Therapy status (None, Failed: IFN, SOF, NS3/5A)** | 4 | 4x24 | = 96 |
| **Co-morbidity: (No, HIV, transplantation,  renal failure)** | 4 | 4x96 | = **384** |

**Supplementary file 3**

Patient profiling of data

TherapySelector aims to answer the clinically relevant question: How often will therapy-specific outcomes (disease remission vs adverse effects) occur in specific patient profiles by various therapy regimens? Therefore, patients who cannot be classified into a patient-profile, who do not have a treatment-specific outcome measurement (those with withdrawal of consent, discontinuation of treatment or lost to follow) or did not follow a specified treatment regimen are excluded; no attempt is made to handle missing data.

TherapySelector uses data from all patients that have started study medication, can be classified into a specific patient profile, have followed a defined therapy regimen and have data on treatment-specific outcome (HCV: virus eradication, SVR12). This approach allows a physician to be informed about the observed results of therapy regimens based on both pre-existing characteristics and therapy regimens specific outcomes.

Patient data from relevant publications are captured into a form that conforms to the TherapySelector database structure. Firstly, data are mapped; secondly, individual patient data are clustered into relevant patient profiles or group data are split into relevant patient profiles.

TherapySelector database structure starts with information on the Publication: references (author, journal, year,) study type (RCT phase 2/3 , RCT phase 4, non-RCT phase 2/3, and real-life non-RCT phase 4) and study name, followed by Patient profile information based on 4 elements: etiology, disease progression, therapy status and comorbidity, then Therapy regimes based on 3 elements: drug or drug combination, dosage and duration, and finally data on Outcome split in 2 categories: 1) therapy specific outcome (success or failure) and 2) non-specific outcome failure (death, discontinuation of therapy, lost-to-follow-up).

Combining patient profiled data

After authorization by a representative of the scientific society for the disease domain (HCV: Netherlands Association for the Study of the Liver), patient profile therapy regimen data (PPDRTD) are imported into the TherapySelector SQL database. The fit of the column data with the column data in the SQL database is checked and if necessary adjusted; thereafter the patient profile therapy regimen dataset is added to the existing data in the TherapySelector’s database.

For each patient profile therapy regimen combination, the absolute numbers of evaluable patients and of patients with a therapy specific outcome (success or failure) are entered in the database. If absolute numbers of a particular patient profile therapy regimen are already present in the database from a previous publication, the new numbers are added, so that the database increasingly reflects the global experience.

The percentage therapy specific success (HCV; SVR12) for each patient profile therapy regimen combination is calculated by dividing the number of patients with therapy specific success by the total number of evaluable patients with that specific patient profile therapy regimen combination in the data base.

This method of data pooling is viewed with suspicion in biostatistics because of the danger of combining heterogeneous populations. By using patient profiles based on all major prognostic and predictive factors, the risk of misleading outcomes is greatly reduced.

**References:**

1 U.S. Food & Drug Administration. Available via <https://www.fda.gov/>.

2 European Medicines Agency. Available via <https://www.ema.europa.eu/en>.

3 Medicines Evaluation Board. Beschikbaar via <https://english.cbg-meb.nl/>.

4 NCBI. MeSH. Beschikbaar via <https://www.ncbi.nlm.nih.gov/mesh> [bezocht op 11-05-2020].

5 Ghany MG, Marks KM, Morgan TR*, et al.* Hepatitis C Guidance 2019 Update: AASLD-IDSA Recommendations for Testing, Managing, and Treating Hepatitis C Virus Infection. *Hepatology*. 2019.

6 Pecoraro V, Banzi R, Cariani E*, et al.* New Direct-Acting Antivirals for the Treatment of Patients With Hepatitis C Virus Infection: A Systematic Review of Randomized Controlled Trials. *J Clin Exp Hepatol*. 2019; 9(4):522-38.

7 Volkmann A, De Bin R, Sauerbrei W*, et al.* A plea for taking all available clinical information into account when assessing the predictive value of omics data. *BMC Med Res Methodol*. 2019; 19(1):162.

8 Sparano JA, Gray RJ, Ravdin PM*, et al.* Clinical and Genomic Risk to Guide the Use of Adjuvant Therapy for Breast Cancer. *N Engl J Med*. 2019; 380(25):2395-405.

9 Kent DM, Rothwell PM, Ioannidis JP*, et al.* Assessing and reporting heterogeneity in treatment effects in clinical trials: a proposal. *Trials*. 2010; 11:85.
